# Role of the lncRNA MALAT1/miR-1 Pathway in Mouse Myocardial Ischemia-Reperfusion Injury

**DOI:** 10.2174/0118715303439119251106075344

**Published:** 2025-11-24

**Authors:** Jiangen Liu, Bin Zhang, Jinlei Li, Zhongfeng Chen, Yangxue Li

**Affiliations:** 1 Department of Cardiology, The Second Hospital of Jilin University, Changchun, Jilin, 130000, China;; 2 Department of VIP Ward, The Second Hospital of Jilin University, Changchun, Jilin, 130000, China;; 3 Guanshu Biotechnology Services (Changchun) Co., Ltd, Changchun, Jilin, 130000, China

**Keywords:** LncRNA MALAT1, hasa-miR-1, myocardial ischemia-reperfusion injury, oxidative stress, apoptosis, cardiovascular pathologies

## Abstract

**Introduction:**

The interplay between LncRNA MALAT1 and hsa-miR-1 plays a crucial role in Myocardial Ischemia-Reperfusion Injury (MIRI), offering insights into the molecular mechanisms underlying cardiovascular pathologies. This study sought to elucidate their regulatory relationship and functional impact on MIRI progression.

**Materials and Methods:**

Using an H9C2 cardiomyocyte cell line subjected to ischemia-reperfusion (I/R) modeling, we analyzed alterations in LncRNA MALAT1 and hsa-miR-1 expression and their downstream effects on apoptosis, reactive oxygen species (ROS) accumulation, and myocardial injury markers.

**Results:**

Our findings demonstrated that siRNA-mediated knockdown of MALAT1 or modulation of hsa-miR-1 (*via* mimics and inhibitors) effectively attenuated oxidative stress and reduced cardiomyocyte apoptosis. Furthermore, *in vivo* experiments using a murine MIRI model corroborated the regulatory roles of MALAT1 and hsa-miR-1, identifying them as potential therapeutic targets for mitigating reperfusion injury.

**Discussion:**

Our findings highlight the importance of the MALAT1/miR-1 axis in MIRI pathogenesis. The observed reduction in ROS and apoptosis upon modulation of these molecules suggests their involvement in key cellular stress responses. These results align with previous studies on lncRNA-miRNA interactions in cardiovascular diseases.

**Conclusion:**

These results not only highlight the significance of the MALAT1/miR-1 axis in MIRI but also propose novel molecular intervention strategies for the treatment of cardiovascular disease.

## 1 INTRODUCTION

Cardiac ischemia-reperfusion (I/R) injury continues to pose significant therapeutic hurdles in managing coronary artery disease, representing the predominant contributor to global cardiovascular disease burden and mortality rates [1]. Although timely reperfusion therapy, such as percutaneous coronary intervention (PCI), effectively restores blood flow to ischemic myocardium, it paradoxically induces additional tissue damage known as reperfusion injury [2].

During the ischemia-reperfusion process, the sudden restoration of oxygen supply triggers a cascade of deleterious events, including mitochondrial dysfunction, calcium overload, and burst production of reactive oxygen species (ROS). These changes disrupt cellular homeostasis, activate inflammatory cascades, and promote apoptosis and necrosis in cardiomyocytes [3, 4]. Thus, myocardial I/R injury is now recognized as a complex pathological process involving oxidative stress, inflammation, mitochondrial permeability transition, and programmed cell death.

Despite decades of investigation, effective strategies to mitigate myocardial I/R injury are still lacking. Understanding how these canonical pathophysiologic mechanisms intersect with molecular regulatory pathways has become an essential step toward developing new cardioprotective strategies.

In recent years, the discovery of regulatory RNAs, encompassing both short microRNAs and long non-coding transcripts, has revealed their crucial functions in cardiac development and disease processes [5, 6]. Characterized by their non-coding nature and length exceeding 200 nucleotides, lncRNAs have emerged as master regulators of cellular processes through their ability to modulate gene expression networks at epigenetic, transcriptional, and post-transcriptional levels [7, 8]. Recent studies have established the regulatory significance of lncRNAs in modulating key pathological processes in myocardial ischemia-reperfusion (I/R) injury, including programmed cell death, reactive oxygen species generation, autophagic flux, and inflammatory responses [9]. lncRNA MALAT1 has increasingly come into focus, stemming from its abnormal expression profiles observed in a range of cardiovascular diseases [10].

The long non-coding RNA MALAT1, exhibiting pan-tissue expression patterns, serves as a master regulator of core cellular phenotypes, including mitotic activity, motility characteristics, and programmed cell death, with significant implications for both oncogenesis and myocardial disorders [11-13]. Recent studies have implicated MALAT1 in the progression of myocardial infarction, cardiac hypertrophy, and ischemic stroke [14, 15]. In myocardial I/R models, MALAT1 expression is typically upregulated, suggesting a potential pathogenic role [16]. Current understanding of MALAT1's pathogenic mechanisms in myocardial injury remains fragmentary.

MicroRNA-1 (miR-1) is one of the most abundant and well-characterized cardiac-specific miRNAs and has been implicated in the regulation of cardiac development, electrophysiological properties, and stress responses [17]. As a key cardioprotective miRNA, miR-1 mitigates I/R-induced cardiac damage through multi-faceted actions, including blockade of apoptotic pathways, scavenging of free radicals, and stabilization of mitochondrial ultrastructure and function [18]. Interestingly, recent studies suggest that miR-1 expression is significantly downregulated in ischemia/reperfusion (I/R) conditions, contributing to cardiomyocyte apoptosis and dysfunction [19].

Given the known functions of both MALAT1 and miR-1 in cardiovascular injury, it is plausible that these two non-coding RNAs may interact in the context of I/R. However, few studies have directly examined whether MALAT1 modulates myocardial injury by regulating miR-1 expression and activity. Considering the critical role of the MALAT1/ miR-1 axis in other disease contexts, such as cancer and neurodegeneration, exploring this interaction in myocardial I/R may provide new insights into non-coding RNA-mediated cardioprotection.

This investigation systematically examined the MALAT1/miR-1 regulatory axis in cardiac ischemia-reperfusion pathology through complementary cellular and animal models. Our working model proposes that MALAT1 exacerbates reperfusion injury by suppressing miR-1, leading to a redox imbalance and apoptotic activation. Experimental approaches included: (1) H9C2 hypoxia-reoxygenation modeling, (2) murine coronary ligation-reperfusion, (3) gain/loss-of-function interventions, and (4) molecular interaction validation. Results demonstrate that silencing MALAT1, coupled with the restoration of miR-1, attenuates oxidative damage and cell death, with luciferase assays confirming their direct interaction. These findings reveal new dimensions of ncRNA crosstalk in cardiac injury and identify druggable targets.

## 2 MATERIALS AND METHODS

### 2.1 Cell Lines and Cell Culture

In this study, two types of cell lines were utilized: rat cardiomyoblasts (H9C2, CM-0089) and human embryonic kidney cells (HEK-293T, CL-0005), both obtained from Procell Life Science & Technology Co., Ltd. (Wuhan, China). The H9C2 cells were maintained in Dulbecco’s Modified Eagle Medium (DMEM; Gibco, USA, 12491015), while HEK- 293T cells were cultured using Minimum Essential Medium (MEM; BDBIO, China, C11095500BT). Each medium was supplemented with 10% fetal bovine serum. All cell cultures were kept in a humidified incubator at 37°C with 5% CO_2_ to ensure optimal growth conditions.

When the cell density in 6-well plates reached 50-60%, the experimental group cells underwent overnight incubation in serum-free medium. Subsequently, they were cultured in minimal essential medium containing 1% FBS, with a final glucose concentration of 30 mM achieved *via* 50% glucose injection to simulate high-glucose conditions for 24 h. Subsequently, the cells were placed in a three-gas incubator maintained at 37°C, where they were subjected to hypoxic conditions (95% nitrogen, 5% carbon dioxide, and 1% oxygen) for four hours. Following this hypoxia induction, the culture plates were transferred to a normoxic environment for a two-hour reoxygenation period.

### 2.2 Cell Transfection

To construct the LncRNA MALAT1 silencing and hsa-miR-1 overexpressing H9C2 cell lines, the small interfering RNA (siRNA) plasmid targeting LncRNA MALAT1 (siR-LncRNA MALAT1) and hsa-miR-1 mimic were constructed. Then, the above plasmids were transfected into H9C2 cell lines using Lipofectamine 2000. Besides, corresponding control siRNA (siR-NC) plasmids were also transfected into H9C2 cells for comparative analysis. The transfection efficiency was detected by RT-qPCR assays. All plasmids were obtained from Youbio (Hunan, China). The sequences of siRNA and miRNA mimics used in this study are listed in Table **1**.

### 2.3 CCK-8

Cell viability was assessed using the Cell Counting Kit-8 (CCK-8; Beyotime, China, C0038). A total of 1000 cells per well were plated into 96-well plates and incubated for 24 hours to allow cell attachment. At designated time intervals (12, 24, and 48 hours), 10 µL of the CCK-8 reagent was added to each well approximately 2 to 4 hours before the end of incubation. Subsequently, the plates were incubated at 37°C, and absorbance at 450 nm was recorded using a microplate reader (BioTek, USA).

### 2.4 RT-qPCR

Total RNA was extracted using Trizol reagent (Ambion, China, 15596018). After incubation at room temperature for 5 min, 200 μL of chloroform was added per 1 mL of Trizol, vortexed for 1 min, incubated for another 5 min, and centrifuged at 12,000 × g for 15 min at 4°C. The aqueous phase was transferred to a new tube, mixed with an equal volume of isopropanol, incubated at room temperature for 10 min, and centrifuged at 12,000 × g for 10 min at 4°C. The RNA pellet was washed with 75% ethanol, centrifuged at 7,500 × g for 5 min, air-dried for 5 min, and dissolved in RNase-free water. RNA concentration and purity were determined spectrophotometrically.

cDNA was synthesized from 1 μg RNA using the PrimeScript™ RT reagent kit (TAKARA, Japan, RR037Q) under the following conditions: 37°C for 15 min and 85°C for 5 s. Quantitative PCR was performed with the TB Green™ Premix Ex Taq™ II kit (TAKARA, Japan, RR820S) using the following cycling program: 95°C for 30 s, followed by 40 cycles of 95°C for 5 s and 60 °C for 30 s. β-actin served as the internal control, and relative expression levels were calculated using the 2^−ΔΔCT^ method.

### 2.5 Western Blotting (WB) Analysis

Protein extraction was carried out by lysing cells in pre-cooled RIPA buffer on ice. The total protein content was quantified using a BCA assay kit (PC0020, Solarbio, China) according to the manufacturer’s instructions. For electrophoresis, 20 μg of protein per sample was loaded onto SDS-PAGE gels and separated based on molecular weight. The resolved proteins were then transferred onto PVDF membranes (88585, Thermo Fisher, USA). Membranes were blocked to minimize nonspecific binding and subsequently incubated with primary antibodies targeting specific proteins of interest. After washing, membranes were treated with horseradish peroxidase (HRP)-conjugated secondary antibodies. Signal detection was performed using an enhanced chemiluminescence (ECL) system (BL520B, Biosharp, China), and bands were visualized accordingly.

### 2.6 Flow Cytometry Assay

To evaluate apoptosis, H9C2 cells were first rinsed twice with chilled PBS and then resuspended in a buffer optimized for flow cytometric assays. The apoptotic status was determined by staining cells with Annexin V-FITC (FXP018, 4A Biotech) and propidium iodide (PI; 550825, BD Biosciences), as directed by the manufacturers. After 5 minutes of incubation in the dark at ambient temperature, fluorescence was detected using the Attune NxT cytometer (Thermo Fisher).

### 2.7 Analysis of the Contents of ROS

Intracellular ROS levels were detected using the fluorescent probe DCFH-DA (10 μM; Beyotime, China, S0033M), which was dissolved in DMEM lacking serum. H9C2 cells were adjusted to a concentration of 1 × 10^6/mL and exposed to the probe for 30 minutes at 37°C under 5% CO_2_ conditions, with gentle mixing performed every 5 minutes to ensure even loading. To visualize mitochondrial oxidative stress and morphology, cells were further co-stained in the dark with 5 μM MitoSOX Red, a specific marker for mitochondrial superoxide, and 100 nM MitoTracker Green, which marks mitochondrial structure. After thorough washing with PBS, nuclear staining was performed using DAPI. Fluorescence imaging was conducted using an IX71 microscope (Olympus, Japan) to capture signals related to ROS.

### 2.8 Determination of Malondialdehyde (MDA), Superoxide Dismutase (SOD), CK, and LDH

H9C2 cells were collected, washed twice with PBS, and treated as indicated. Cell culture supernatants were used for CK and LDH assays, while cell pellets were lysed by ultrasonication on ice and centrifuged at 12,000 × g for 10 min.

For myocardial tissue, ventricular samples were minced, homogenized in ice-cold lysis buffer, and centrifuged at 12,000 × g for 10 min. The supernatants were collected for MDA, SOD, CK, and LDH measurements.

MDA was measured using the thiobarbituric acid (TBA) method (532 nm), and SOD activity was determined using the xanthine oxidase method (550 nm), following the kit instructions (Nanjing Jiancheng Bioengineering Institute, Cat. Nos. A003-2-2, A001-3-1). CK and LDH activities were measured in supernatants using commercial kits (Cat. Nos. A032-1-1, A020-2-2).

### 2.9 Bioinformatics

Possible interaction regions between hsa-miR-1 and the long non-coding RNA MALAT1 were identified through bioinformatic prediction using the StarBase v3.0 platform (https://starbase.sysu.edu.cn/). The LncRNA–miRNA interaction module was used, and “MALAT1” was entered as the lncRNA of interest, with “hsa-miR-1-3p” selected as the candidate miRNA. Default parameters were applied, including support for CLIP-Seq data. The predicted complementary binding sequences and binding regions were recorded and used for the subsequent construction of a dual-luciferase reporter assay vector.

### 2.10 Dual-Luciferase Reporter Gene Assay

To investigate the potential binding relationship between hsa-miR-1 and MALAT1, sequences containing either the wild-type or mutated miR-1 binding regions of MALAT1 were subcloned into the pmirGLO luciferase reporter plasmid (Promega, USA). HEK293T cells were seeded in 24-well culture plates and maintained until they reached approximately 70%–80% confluency. Lipofectamine 2000 (Thermo Fisher Scientific, USA) was used to deliver the reporter constructs into the cells *via* co-transfection. Following a 24-hour incubation, luciferase expression was assessed using the Dual-Luciferase Reporter Assay Kit (Promega).

### 2.11 Construction of Animal Models *In Vivo*

Twenty-five male C57BL/6 mice (6–8 weeks old, 20–30 g) were obtained from Vital River Laboratory Animal Technology Co., Ltd. (Jiaxing, China). Before experimentation, mice were housed under controlled conditions (22 ± 2°C, 12 h light/dark cycle) for one week to acclimate, with food and water available ad libitum.

After acclimatization, animals were randomly divided into five groups (n = 5 per group): (1) sham-operated controls (thoracotomy without LAD ligation), (2) LncRNA MALAT1-silenced, (3) negative control siRNA, (4) hsa-miR-1 overexpressing, and (5) hsa-miR-1 suppressed groups. Except for the sham group, all other groups were subjected to myocardial ischemia/reperfusion (I/R) surgery.

The MI/RI model was established following standard protocols. After anesthesia with sodium pentobarbital (30 mg/kg, i.p.), mice were intubated and mechanically ventilated. A standard limb-lead electrocardiogram (ECG) was continuously recorded throughout the procedure using a small-animal physiological monitoring system (BL-420S, Chengdu Taimeng, China). Myocardial ischemia was confirmed by ST-segment elevation on ECG, visible pallor of the ventricular wall, and reduced contractility. The left anterior descending (LAD) coronary artery was occluded for 30 min using 6-0 silk sutures, followed by reperfusion for 120 min after suture release.

All constructs were administered *via* tail vein injection 7 days before I/R surgery using an *in vivo* transfection reagent. Ten days after reperfusion, mice were euthanized, and hearts were excised for analysis. Each heart was transversely sectioned into multiple slices: a portion was used for TTC staining to assess infarct size, while the remaining tissue from the same heart was preserved for histological staining, RNA extraction, and protein analysis.

### 2.12 TTC Staining

To quantify myocardial infarction, heart tissues were subjected to TTC staining. Following euthanasia, excised hearts were promptly placed at −80°C for 20 minutes to aid in subsequent slicing. The frozen tissues were then transversely sectioned into 2 mm thick slices using a precision tissue matrix. Tissue slices were immersed in 1% TTC working solution and maintained at 37°C for 15 minutes in the absence of light. After staining, samples were fixed in 4% paraformaldehyde to enhance color differentiation. The pale (non-stained) regions indicating infarction were measured with ImageJ software and calculated as a proportion of the entire ventricular area.

### 2.13 Pathological Staining

Tissue samples were initially fixed in 10% neutral formalin solution for 24 hours. Subsequently, they underwent stepwise dehydration through a graded ethanol series, starting at 70% and progressing to 80% and 90% concentrations. Following dehydration, samples were treated with xylene in two changes to achieve complete clearing before being embedded in molten paraffin. After embedding, thick sections were cut, mounted on slides, and dried. For staining, sections underwent xylene dewaxing, graded ethanol hydration (100% to 70%), hematoxylin staining (5-10 min), 1% HCl-ethanol differentiation, bluing in tap water/ammonia, and eosin counterstaining (1-3 min). Finally, sections were dehydrated in ethanol, dried, and examined under a microscope.

### 2.14 Statistical Analysis

All statistical analyses were performed using GraphPad Prism version 6.0 (USA, SCR_002798). Data normality was assessed using the Shapiro–Wilk test. For normally distributed data, comparisons between two groups were analyzed using an unpaired Student’s t-test, while multiple group comparisons were conducted by one-way analysis of variance (ANOVA) followed by Tukey’s post hoc test. When data did not meet normality assumptions, nonparametric tests (Mann–Whitney U test or Kruskal–Wallis test) were applied. Each dataset represents the mean of at least three independent biological replicates, and results are expressed as mean ± SD. A *p*-value < 0.05 was considered statistically significant (*p* < 0.05, *p* < 0.01).

## 3 RESULTS

### 3.1 lncRNA MALAT1 and miR-1 Expression in I/R Injured H9c2 Cells

To investigate the molecular response to ischemic injury, H9C2 cardiomyocytes were subjected to 24 hours of hypoxic conditions followed by 2 hours of reoxygenation, simulating ischemia-reperfusion (I/R) conditions. In comparison to the normoxia group, cells exposed to ischemia/reperfusion exhibited a substantial elevation in MALAT1 transcript levels, whereas hsa-miR-1 expression was significantly diminished, as determined by RT-qPCR analysis (Fig. **1A**), suggesting involvement of the MALAT1/miR-1 axis in I/R-induced injury.

Functionally, I/R treatment led to a significant increase in apoptosis and a reduction in cell viability (Fig. **1B** and **1C**). I/R also induced oxidative stress, evidenced by elevated mitochondrial ROS levels (MitoSOX staining), decreased SOD activity, and increased MDA content (Fig. **1D**-**F**). Western blot results showed upregulation of Bax, caspase-3, and myocardial injury markers CK and LDH, along with downregulation of Bcl-2, indicating activation of apoptotic pathways and substantial cellular damage under I/R conditions (Fig. **1G**).

Together, these results suggest that the MALAT1/miR-1 axis may be involved in I/R-induced cardiomyocyte injury, potentially by promoting oxidative stress and apoptosis.

### 3.2 The effects of LncRNA MALAT1 silencing and hsa-miR-1 overpressing on H9C2 cells with I/R injury

To investigate the potential roles of LncRNA MALAT1 and hsa-miR-1 in cellular injury caused by ischemia/reperfusion, H9C2 cardiomyocytes were subjected to transfection with siRNA targeting MALAT1 or a synthetic miR-1 analog. The efficiency of these transfections was confirmed through RT-qPCR (Fig. **2A** and **B**). Functional experiments demonstrated that knocking down MALAT1 or upregulating hsa-miR-1 notably enhanced SOD activity while decreasing MDA content relative to the I/R model group, suggesting a mitigation of oxidative stress (Fig. **2C** & **D**). In parallel, apoptosis rate and ROS production were also significantly reduced under these conditions (Fig. **2E** and **F**). Notably, co-transfection with an hsa-miR-1 inhibitor reversed these protective effects.

WB analysis consistently showed that reducing MALAT1 levels or increasing hsa-miR-1 expression significantly lowered the amounts of pro-apoptotic proteins Bax and caspase-3, along with myocardial injury indicators CK and LDH, while promoting the expression of the anti-apoptotic protein Bcl-2 (Fig. **2G**). These effects were also reversed by the hsa-miR-1 inhibitor, supporting the hypothesis that MALAT1 promotes I/R-induced cardiomyocyte injury, at least in part, *via* the negative regulation of hsa-miR-1.

### 3.3 Validation of the Targeting Relationship between LncRNA MALAT1 and hsa-miR-1

To validate the direct interaction between LncRNA MALAT1 and hsa-miR-1, we used the StarBase prediction platform (https://starbase.sysu.edu.cn) to identify putative miRNA binding sites within the MALAT1 transcript (Fig. **3A**). Based on the predicted seed sequence, WT and mut MALAT1 reporter constructs were generated for dual-luciferase reporter assays.

As shown in Fig. (**3B**), Dual-luciferase assays demonstrated that hsa-miR-1 overexpression substantially suppressed the reporter activity of WT-MALAT1, while showing no inhibitory effect on the Mut binding site construct. These results confirm that MALAT1 directly binds to hsa-miR-1 *via* the predicted site to regulate miR-1 activity.

### 3.4 The Effect of LncRNA MALAT1 and hsa-miR-1 in I/R Injury was Validated *In Vivo*

To confirm the functional role of LncRNA MALAT1 and hsa-miR-1 *in vivo*, a mouse myocardial I/R model was established. Mice were intravenously injected with vectors encoding si-MALAT1, hsa-miR-1 mimics, or inhibitors to modulate gene expression. They were randomly assigned to five cohorts: Sham, I/R, si-MALAT1, miR-1 mimic, and miR-1 inhibitor. RT-qPCR of heart tissue showed elevated MALAT1 and reduced hsa-miR-1 levels in the I/R group, consistent with *in vitro* results (Fig. **4A**).

Serum levels of myocardial injury markers, including CK and LDH, were significantly elevated in the model group, and were markedly reduced by MALAT1 silencing or hsa-miR-1 overexpression. At the same time, administration of hsa-miR-1 inhibitor reversed these protective effects (Figs. **4B** and **C**). Similarly, oxidative stress analysis showed that MDA levels were reduced and SOD activity was elevated in the si-MALAT1 and hsa-miR-1 mimic groups, further supporting their antioxidant roles (Figs. **4D** and **E**).

Histological analysis by HE and TTC staining revealed that MALAT1 knockdown and hsa-miR-1 overexpression alleviated myocardial structural damage and reduced infarct size (Figs. **4F** and **G**). TUNEL assays revealed markedly decreased apoptotic rates in cardiomyocytes following treatment, an effect that was completely abolished by hsa-miR-1 inhibition (Fig. **4H**).

Collectively, these *in vivo* observations further verify that silencing of MALAT1 and upregulation of hsa - miR - 1 confer protection against myocardial I/R injury. This protective effect probably occurs *via* the modulation of oxidative stress, apoptosis, and the release of myocardial enzymes.

## 4 DISCUSSION

Myocardial I/R injury, a major pathological underpinning of ischemic heart diseases, is characterized by heightened oxidative stress, inflammation, and cardiomyocyte apoptosis. Growing evidence highlights how non-coding RNAs, particularly long non-coding RNAs (lncRNAs) and microRNAs (miRNAs), modulate these pathological processes [20, 21]. Although previous studies have suggested that MALAT1 and miR-1 are involved in myocardial injury, their direct molecular interplay and combined effects on oxidative stress and apoptosis have not been previously elucidated.

In this study, we provide the first evidence that LncRNA MALAT1 directly targets and suppresses hsa-miR-1 to aggravate cardiomyocyte injury during I/R, thereby establishing a novel MALAT1/hsa-miR-1 regulatory axis that orchestrates oxidative stress and apoptosis both *in vitro* and *in vivo*. This mechanistic link reveals a new layer of ncRNA-based regulation underlying myocardial ischemia-reperfusion injury.

We first established an H/R model in H9C2 cells to mimic myocardial ischemia/reperfusion (I/R) injury. The results revealed significant upregulation of MALAT1 and downregulation of hsa-miR-1 in the I/R group, suggesting a potential regulatory axis between the two. Functionally, H/R treatment induced marked increases in cardiomyocyte apoptosis, mitochondrial ROS generation, and MDA levels, accompanied by reductions in SOD activity and cell viability. These phenotypes are consistent with previous studies, which indicate that oxidative stress is a primary driver of cardiomyocyte dysfunction following ischemia-reperfusion (I/R) insult.

To dissect the regulatory relationship between MALAT1 and hsa-miR-1, we performed loss- and gain-of-function experiments in H9C2 cells. Silencing MALAT1 or overexpressing hsa-miR-1 significantly alleviated oxidative stress, reduced apoptosis, and restored cell viability, as evidenced by increased SOD levels, decreased ROS and MDA levels, and downregulation of apoptotic markers including Bax and caspase-3. Importantly, these protective effects were reversed upon co-treatment with hsa-miR-1 inhibitor, supporting the notion that MALAT1 may negatively regulate hsa-miR-1.

Dual-luciferase reporter assays further verified the direct binding interaction between MALAT1 and hsa-miR-1. Hsa-miR-1 mimics notably suppressed the luciferase activity of the wild-type MALAT1 construct, whereas no significant alteration was observed in the mutant construct lacking the predicted binding site. These findings validate the post-transcriptional regulatory relationship between MALAT1 and hsa-miR-1.

*In vivo* experiments using a mouse myocardial I/R model provided additional evidence supporting the functional relevance of the MALAT1/hsa-miR-1 axis. Consistent with the *in vitro* findings, I/R injury in mice resulted in upregulation of MALAT1 and suppression of hsa-miR-1 in myocardial tissue. Silencing MALAT1 or overexpressing hsa-miR-1 significantly reduced serum levels of cardiac injury markers (CK and LDH), attenuated oxidative stress (as indicated by increased SOD and decreased MDA), and improved cardiac histopathology and infarct size. Furthermore, TUNEL staining revealed a substantial decrease in cardiomyocyte apoptosis in the si-MALAT1 and hsa-miR-1 mimic groups. These protective effects were abrogated by the administration of a hsa-miR-1 inhibitor, further confirming the functional involvement of the MALAT1/hsa-miR-1 axis in myocardial protection.

(Fig. **5**) Collectively, our findings define a novel mechanistic pathway in which LncRNA MALAT1 acts as a competing endogenous RNA (ceRNA) that sequesters miR-1, leading to enhanced oxidative stress and apoptosis in cardiomyocytes. The identification of this MALAT1/hsa-miR-1 axis provides new insight into ncRNA-mediated myocardial injury and expands the understanding of the post-transcriptional regulatory network involved in I/R pathology.

In summary, this study identifies and functionally validates the MALAT1/hsa-miR-1 signaling axis as a novel regulator of myocardial ischemia/reperfusion (I/R) injury. By linking MALAT1 to miR-1-mediated oxidative and apoptotic pathways, we uncover a potential molecular target for cardioprotection. Future studies should further investigate the upstream regulators of MALAT1 expression under I/R stress, as well as the downstream targets of miR-1 that mediate its cardioprotective effects.

## CONCLUSION

In brief, LncRNA MALAT1 amplifies myocardial I/R injury *via* downregulation of hsa-miR-1, which promotes oxidative stress and apoptosis in cardiomyocytes. Silencing MALAT1 or overexpressing hsa-miR-1 effectively alleviated I/R-induced cellular injury, reduced ROS accumulation, improved antioxidant capacity, and suppressed apoptosis both *in vitro* and *in vivo*. Dual-luciferase reporter assays confirmed the direct interaction between MALAT1 and hsa-miR-1, highlighting the MALAT1/miR-1 regulatory axis as a key contributor to myocardial I/R injury. These results provide new insights into how non-coding RNAs affect cardiac damage and suggest that targeting the MALAT1/miR-1 pathway could be a potential therapeutic approach to mitigate myocardial injury caused by I/R. Further studies are warranted to explore upstream regulators and downstream effectors involved in this axis.

## AUTHORS’ CONTRIBUTIONS

The authors confirm their contribution as follows: J.L. contributed to the study design, manuscript preparation, and data interpretation. B Z participated in study design, data analysis, and manuscript writing. J L was involved in study design, data collection, and statistical analysis. Z C contributed to study planning, data collection, and data interpretation. Y L participated in study design, literature review, and funding acquisition.

## Figures and Tables

**Fig. (1):**
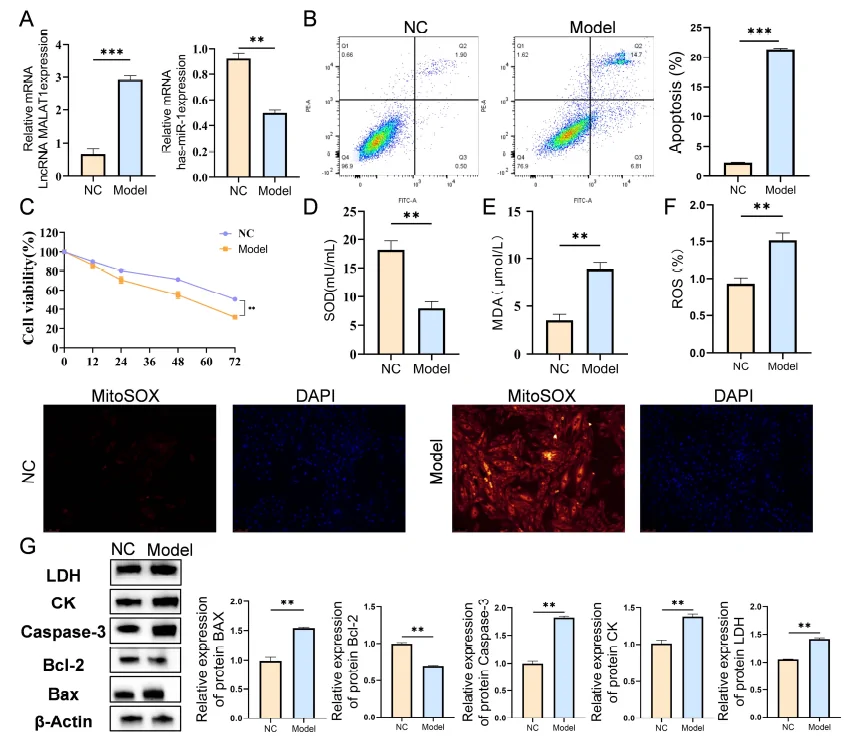
lncRNA MALAT1 and miR-1 expression in ischemia/reperfusion (I/R) -injured H9c2 cells. (**A**) The expression levels of LncRNA MALAT1 and hsa-miR-1 transcripts in H9C2 cells were analyzed by quantitative reverse transcription PCR. (**B**) Apoptotic cell populations were quantified using flow cytometry. (**C**) Cellular metabolic activity, reflecting viability, was evaluated with the CCK-8 assay. (**D** & **E**) The concentrations of SOD (**D**) and MDA (**E**) in H9C2 cells were measured using enzyme-linked immunosorbent assay (ELISA) kits. (**F**) Mitochondrial reactive oxygen species (ROS) production was visualized through immunofluorescent staining techniques. (**G**) Protein expression levels of BAX, Bcl-2, Caspase-3, CK, and LDH were detected *via* WB. Three independent experiments were performed for the assays. The statistical significance level was set at 0.05, with * corresponding to a *p*-value < 0.05, ** corresponding to a *p*-value < 0.01, and *** corresponding to a *p*-value < 0.001.

**Fig. (2):**
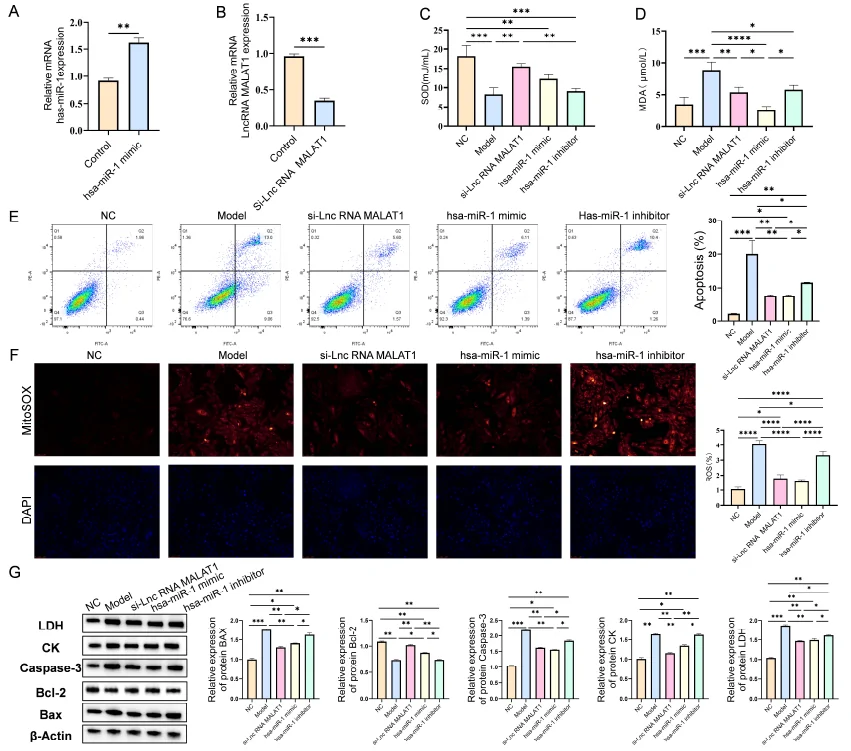
The effects of LncRNA MALAT1 silencing and hsa-miR-1 overpressing on H9C2 cells with I/R injury. H9C2 cells with LncRNA MALAT1 silencing and hsa-miR-1 overexpression were established. (**A** and **B**) The success of gene silencing and overexpression in H9C2 cells was confirmed by quantitative RT-PCR analysis. (**C** and **D**) Intracellular SOD (**C**) and MDA (**D**) contents were determined using commercial ELISA kits. (**E**) Apoptotic cell proportions were analyzed using flow cytometry. Mitochondrial oxidative stress levels were visualized *via* immunofluorescent staining to detect ROS accumulation. (**F**) The protein levels of BAX, Bcl-2, Caspase-3, CK, and LDH were examined by Western blotting to evaluate apoptosis and myocardial injury markers. Three independent experiments were performed for the assays. The statistical significance level was set at 0.05, with * corresponding to a *p*-value < 0.05, ** corresponding to a *p*-value < 0.01, and *** corresponding to a *p*-value < 0.001.G

**Fig. (3):**
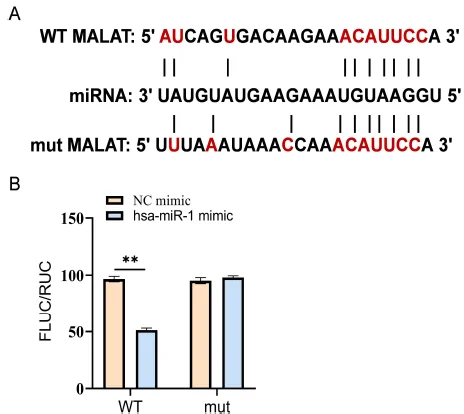
Validation of the targeting relationship between LncRNA MALAT1 and hsa-miR-1. (**A**) The potential binding regions of hsa-miR-1 within the LncRNA MALAT1 sequence were computationally identified through the Starbase bioinformatics platform. (**B**) A dual-luciferase reporter system was employed to experimentally confirm the direct interaction between MALAT1 and hsa-miR-1.

**Fig. (4):**
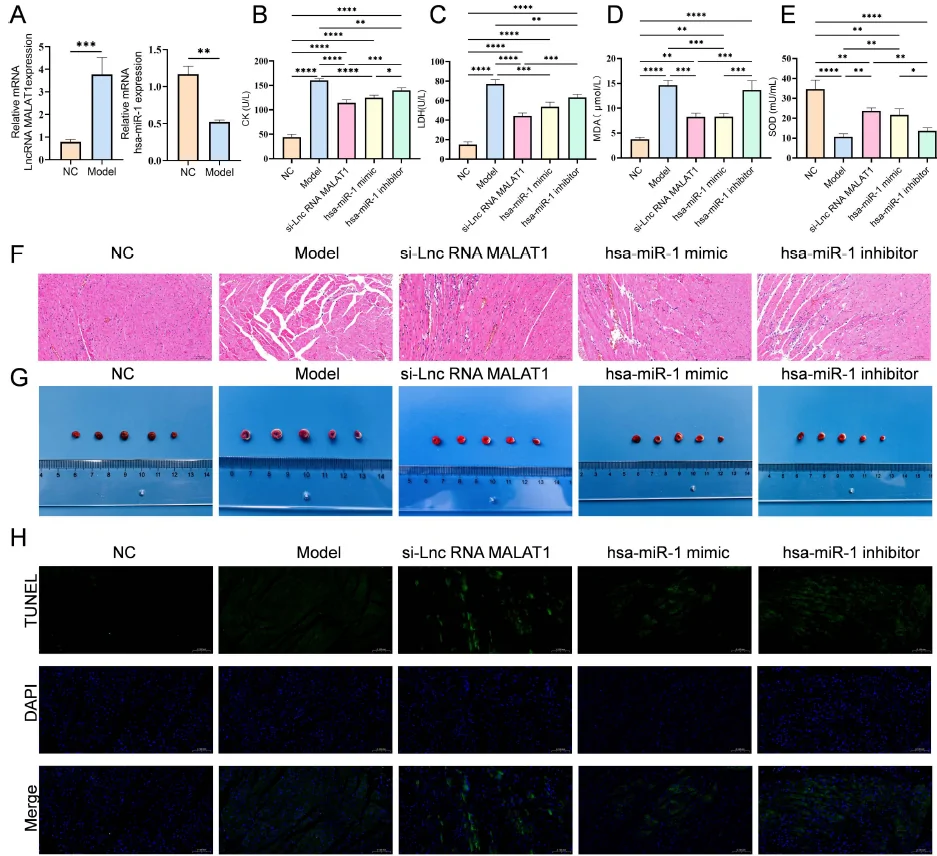
The effect of LncRNA MALAT1 and hsa-miR-1 in I/R injury was validated *in vivo.* (n=5). (**A**) The mRNA expression levels of LncRNA MALAT1 and hsa-miR-1 in myocardial tissues were measured using RT-qPCR. (B-E) The concentrations of CK (**B**), LDH (**C**), MDA (**D**), and SOD (**E**) in myocardial tissues were quantified with ELISA kits. (**F**) Histological alterations in myocardial tissues were examined through hematoxylin and eosin (HE) staining. (**G**) Representative images of cardiac TTC staining. (**H**) Representative images of TUNEL staining in myocardial tissues. Three independent experiments were performed for the assays. The statistical significance level was set at 0.05, with * corresponding to a *p*-value < 0.05, ** corresponding to a *p*-value < 0.01, and *** corresponding to a *p*-value < 0.001.

**Fig. (5):**
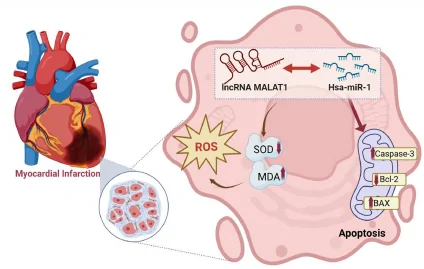
Schematic illustration of the LncRNA MALAT1/hsa-miR-1 axis mediating myocardial ischemia-reperfusion injury *via* regulating oxidative stress and apoptosis. LncRNA MALAT1 negatively regulates hsa-miR-1 *via* direct binding. In myocardial ischemia-reperfusion injury, this axis imbalance leads to ROS accumulation, SOD reduction, MDA elevation (oxidative stress), and altered expression of Bax, Caspase-3, Bcl-2 (apoptosis), ultimately exacerbating myocardial infarction.

**Table 1:** The sequences of siRNAs.

**Name**	**Sense**	**Antisense**
siR-NC	UUCUCCGAACGAGUCACGUTT	ACGUGACUCGUUCGGAGAATT
siRNA MALAT1	GGCUUAUACUCAUGAAUCUTT	UUCUCCGAACGUGUCACGUTT
hsa-miR-1 mimic	UGGAAUGUAAAGAAGUAUGUAU	AUACAUACUCUUUAUCAUUCCA
miR-NC mimic	UUCUCCGAACGUGUCACGUTT	ACGUGACACGUUCGGAGAATT

## Data Availability

All data generated or analyzed during this study are shown within this article.

## LIST OF ABBREVIATIONS

MIRI: Myocardial Ischemia-Reperfusion Injury
I/R: Ischemia-Reperfusion
ROS: Reactive Oxygen Species
siRNA: Small Interfering RNA
RT-qPCR: Reverse Transcription Quantitative Polymerase Chain Reaction
CCK-8: Cell Counting Kit-8
WB: Western Blotting
HRP: Horseradish Peroxidase
ECL: Enhanced Chemiluminescence
DCFH-DA: 2',7'-Dichlorodihydrofluorescein Diacetate
MDA: Malondialdehyde
SOD: Superoxide Dismutase
CK: Creatine Kinase
LDH: Lactate Dehydrogenase
ANOVA: Analysis of Variance
TTC: 2,3,5-Triphenyltetrazolium Chloride
FITC: Fluorescein Isothiocyanate
PI: Propidium Iodide
HE: Hematoxylin and Eosin
WT: Wild-Type
Mut: Mutant
NC: Negative Control
miRNA: MicroRNA
lncRNA: Long Non-Coding RNA
cDNA: Complementary DNA
FBS: Fetal Bovine Serum
DMEM: Dulbecco’s Modified Eagle Medium
MEM: Minimum Essential Medium
PBS: Phosphate-Buffered Saline
BCA: Bicinchoninic Acid
LAD: Left Anterior Descending Coronary Artery
TUNEL: Terminal Deoxynucleotidyl Transferase dUTP Nick-End Labeling
PCI: Percutaneous Coronary Intervention
